# Simultaneous molecular docking of different ligands to His_6_-tagged organophosphorus hydrolase as an effective tool for assessing their effect on the enzyme

**DOI:** 10.7717/peerj.7684

**Published:** 2019-09-12

**Authors:** Aysel Aslanli, Elena Efremenko

**Affiliations:** 1 Faculty of Chemistry, Lomonosov Moscow State University, Moscow, Russia; 2 N.M. Emanuel Institute of Biochemical Physics RAS, Moscow, Russia

**Keywords:** Antibiotic, N-acyl homoserine lactone, Hexahistidine-tagged organophosphorus hydrolase, Lactonase activity, Molecular docking

## Abstract

**Background:**

Enzymatic hydrolysis of N-acyl homoserine lactones (AHLs), which are signaling molecules responsible for the development of antibiotic resistance in gram-negative bacteria, is a potential solution to overcoming antibiotic resistance problem. It has been established that hexahistidine-tagged organophosphorus hydrolase (His_6_-OPH) exhibits lactonase activity against a number of AHLs and that the combined application of His_6_-OPH with β-lactam antibiotics leads to an increase in the efficiency of the action of both the enzyme and antibiotics. The use of computational methods can be an effective way to search for and select from the known antibiotics to find the most rational “partners” for combining with this enzyme and creating effective antibacterial agents with a dual (lactonase and antibacterial) functional activity.

**Methods:**

In this study, by using AutoDock Vina and Gromacs softwares the molecular docking and the molecular dynamics methods were adopted to simulate models of puromycin, ceftiofur, and/or AHLs docked to the surface of a dimer molecule of His_6_-OPH and to study their binding properties. GABEDIT and GAMESS-US packages were used to generate and simulate electron densities of docked AHLs.

**Results:**

Interactions of N-butyryl-DL-homoserine lactone (C4-HSL), N-(3-oxooctanoyl)-L-homoserine lactone (C8-HSL) and N-(3-oxododecanoyl)-L-homoserine lactone (C12-HSL) with His_6_-OPH dimer active sites in the presence of puromycin and ceftiofur were simulated and studied. The possible intersection of long-chain AHLs with antibiotic molecules in the active sites of the enzyme was revealed. The binding energies of antibiotics and AHLs with the His_6_-OPH surface were estimated. Statistically significant differences (*p* = 0.003) were observed between the values calculated for both C4-HSL and C12-HSL, whereas there were no statistically significant differences between the values of the other groups (*p* ≥ 0.100). The binding energies of AHLs with His_6_-OPH were slightly higher as compared with the binding energies of antibiotics with the enzyme. The dynamics of the most probable models obtained from docking were investigated. RMSD and RMSF analysis of His_6_-OPH-AHL complexes in the absence and presence of antibiotics were performed. The interaction energy values of antibiotics and AHLs with the His_6_-OPH were assessed. Significant increase of the AHLs steadiness in enzyme-substrate complexes in the presence of antibiotics was revealed. The interaction between His_6_-OPH and C12-HSL was established as thermodynamically more favored.

**Conclusions:**

It has been established that the studied antibiotics puromycin and ceftiofur steady the enzyme-substrate complexes, but at the same time lead to a decrease in the long-chain AHL-hydrolytic activity of His_6_-OPH in such a combination as compared to a native enzyme, and, therefore, it should be taken into account when creating a therapeutic composition based on combining antibiotics with His_6_-OPH.

## Introduction

To date, bacterial diseases are among the most common and hazardous to humans. While some bacteria play a vital role in ecology, others can become sources of dangerous human diseases, including fatal ones. Today, a number of antimicrobial agents are used to fight bacterial pathogenicity, the most important of which are various antibiotics (Von Döhren, 2004). The wide dissemination of pathogenic microorganisms has recently led to an uncontrolled increase in the amount of antibiotics consumed, which, in turn, has caused the development of bacterial strains resistant to the action of antimicrobial agents. The development of antibiotic-resistant pathogens has resulted in the loss of the action effectiveness of some antibiotics applied in practice and the subsequent need to increase their usage doses (Fischbach & Walsh, 2009). As a result, antibiotic resistance has become one of the major problems of our time, requiring the development of effective approaches to address the issue (Ventola, 2015).

The resistance mechanisms of most bacterial species are well studied today (Mah & O’toole, 2001). The Quorum Sensing (QS), mechanism describes the ability of bacterial cells to interact with each other within the same population to develop highly resistant antibiotic associations (Ng & Bassler, 2009). It is known that the majority of pathogenic gram-negative bacteria use signal molecules, acyl homoserine lactones (AHLs), as QS inducers. The synthesis of different AHLs is typical for various bacteria and the acyl chain length of AHLs usually ranges from C4 to C18 (Whitehead et al., 2001; Miller & Bassler, 2001). For example, common bacterial pathogens such as *Pseudomonas aeruginosa*, *Burkholderia cepacia*, and *Yersinia enterocholitica* secrete C12-, C8-, and C6- containing AHLs as QS signal molecules, respectively (Parsek & Greenberg, 2000).

It is supposed that one of the effective methods to overcome bacterial resistance may be the enzymatic degradation of the QS signal molecules of gram-negative bacteria. There are two basic groups among the known enzymes capable of hydrolyzing AHLs: (1) lactonases which eliminate ester bond in lactones that results in the lactone ring opening, and (2) acylases which hydrolyze an amide bond between the lactone ring and the acyl chain, that results in the production of homoserine lactone and fatty acids (Chen et al., 2013). The lactonases catalyzing the lactone ring cleavage in AHLs are of particular interest. Hexahistidine-tagged organophosphorus hydrolase (His_6_-OPH) is one such enzyme. It was previously shown that His_6_-OPH, in addition to its high catalytic activity in the process of hydrolysis of a number of organophosphorous compounds, also has lactonase activity against a number of AHLs (Maslova et al., 2017a; Maslova et al., 2017b). It has been established that combinations of His_6_-OPH with β-lactam antibiotics provide an increase in the efficiency of action of both the enzyme and antibiotics (Maslova et al., 2017a; Maslova et al., 2017b; Aslanli, Lyagin & Efremenko, 2018). These results reinforce the interest in studying the possibility of combining His_6_-OPH with other antibiotics characterized by different chemical properties and a spectrum of bacterial targets (Azzam & Algranati, 1973; Salmon, Watts & Yancey Jr, 1996). Recently, it was shown that in the presence of the enzyme, there is an observed decrease in the minimum inhibitory concentrations of the puromycin and ceftiofur against *Pseudomonas sp.* cells simultaneously producing different AHLs (Maslova et al., 2018). Therefore, it is of high scientific interest to determine the effect of these antibiotics on the catalytic activity of His_6_-OPH.

**Figure 1:**
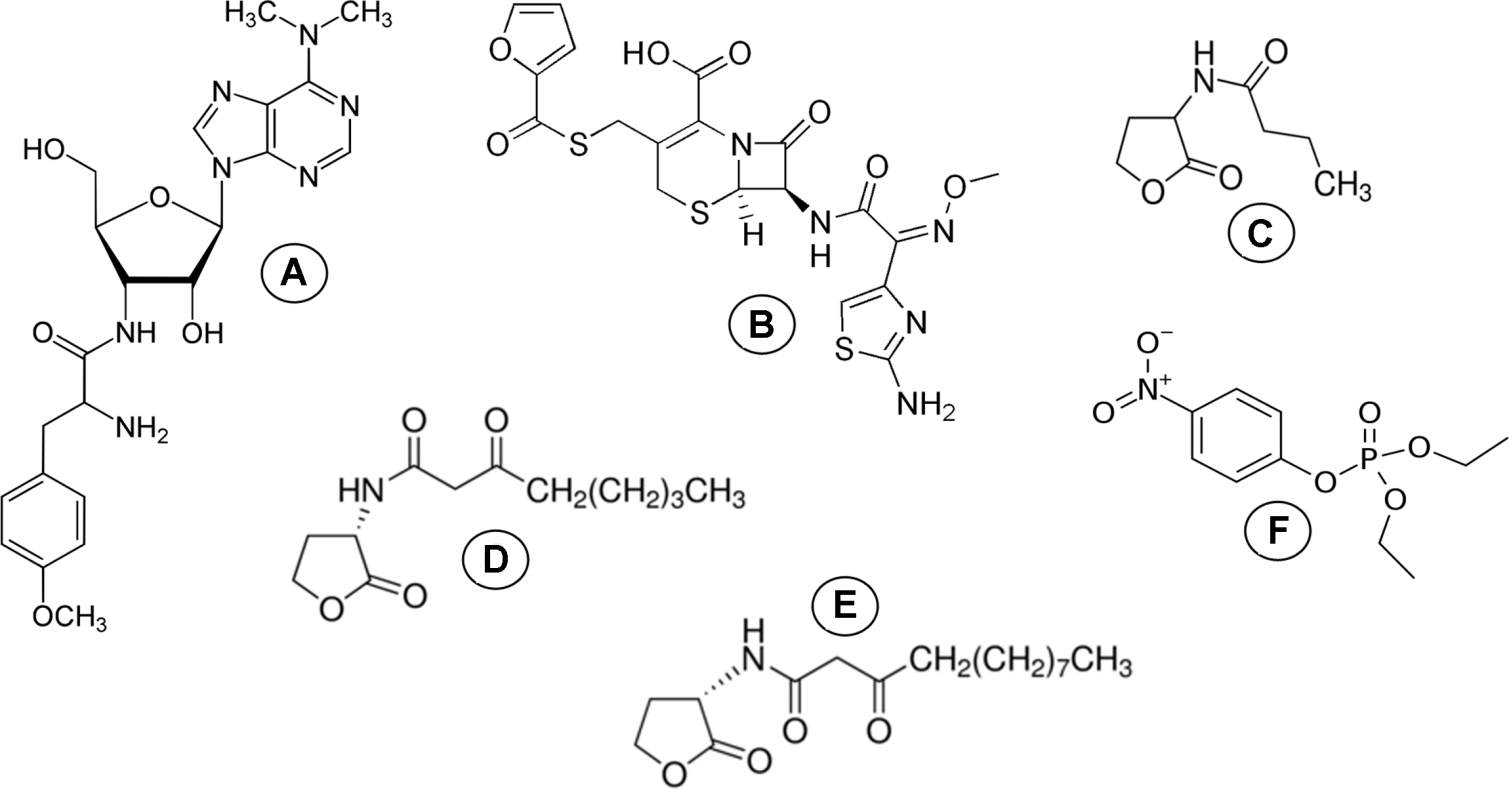
Molecular structure of ligands. Molecular structure of (A) puromycin, (B) ceftiofur, (C) C4-HSL, (D) C8-HSL, (E) C12-HSL and (F) paraoxon.

The previous study (Aslanli, Lyagin & Efremenko, 2018) demonstrated and experimentally confirmed the possible application of molecular docking as a method for the simulation of models of the concurrently binded ligands to the single active site of the His_6_-OPH enzyme and developing its combined preparations with β-lactam antibiotics. However, there is no available information about the dynamics between the protein and ligands in such models of combined biocatalytic systems. So, the originality of the present study is the use of computational methods to better understand the simultaneous interactions between His_6_-OPH and different ligands being substrates or stabilizers. In this regard, the methods of molecular docking and molecular dynamics were applied in this work to simulate models of His_6_-OPH in combination with antibiotics or/and AHLs to estimate the possible expanding the spectrum of antibiotics suitable for combining with the enzyme and evaluate its lactonase activity. These studies were carried out by using antibiotics such as puromycin and ceftiofur and different AHLs-like enzyme substrates (N-butyryl-DL-homoserine lactone (C4-HSL), N-(3-oxooctanoyl)-L-homoserine lactone (C8-HSL) and N-(3-oxododecanoyl)-L-homoserine lactone (C12-HSL)) naturally synthesized by various bacteria under real conditions (Fig. 1).

## Materials & Methods

### Molecular docking

His_6_-OPH dimer was modeled using the modified crystallographic structure of organophosphorus hydrolase (RSCB PDB number 1QW7) (Fig. S1) (Lyagin & Efremenko, 2018).

ChemBioDraw software (ver. 12.0, CambridgeSoft) was used to create the ligands structures (AHLs and antibiotics). Then ChemBio3D was used to perform energy with force field MM2. Lastly, AutoDockTools (as part of MGLTools ver. 1.5.6, available at http://mgltools.scripps.edu/) (Morris et al., 2009) was applied to obtain the ligands structures in PDBQT format (PDB format with partial charges and atom types) from files in PDB format (protein data bank). The Gasteiger-Marsili method was used to calculate atomic charges.

Charge distribution at the surface of His_6_-OPH at pH 7.5 and 10.5 was calculated using adaptive Poisson–Boltzmann solver (APBS) and PDB2PQR servers (ver. 1.4.2.1 and 2.1.1, respectively, available at http://www.Poissonboltzmann.org/) with PARSE force field (Dolinsky et al., 2007). The crystallographic structure of the OPH dimer (PDB number 1QW7) modificated by His_6_-tag (Lyagin & Efremenko, 2018) was used as a basis.

AutoDock Vina (ver. 1.1.2, available at http://vina.scripps.edu/) (Trott & Olson, 2010) was applied to perform the docking of antibiotics (Figs. S2, S3) and AHLs to the surface of His_6_-OPH dimer molecule at pH 7.5 and 10.5 on a desktop computer equipped with Intel Pentium Dual-Core CPU E5400 2.7 GHz and 3 GB of available memory. In short, the grid box, which size was chosen in accordance with the size of the enzyme molecule surface (for details see PDB number 1QW7), was approximately centered on the center of mass of the His_6_-OPH dimer. To perform calculations the program options were set as default.

The GAMESS-US packages (Schmidt et al., 1993) under the restricted Hartree–Fock calculation with Huzinaga’s three gaussian minimal basis set were used to simulate the electron densities of the docked AHLs. Electron density (.CUBE) files (Allouche, 2011) were generated using GABEDIT software. Finally, structures were visualized with PyMOL Molecular Graphics System (ver. 1.7.6, Schrödinger, LLC).

### Molecular dynamics simulations

For further study and better understanding interactions in protein-ligand complexes MD simulations were used (Hansson, Oostenbrink & van Gunsteren, 2002). Gromacs software was used to carry out MD simulations were (Abraham et al., 2015) Using the TIP3P water model, CHARMM36 force field and CGenFF server coordinates and topology files of protein and ligands were generated (Jorgensen et al., 1983; Vanommeslaeghe et al., 2010). Then, protein–ligand complexes were built and their topology files were constructed by editing the topology files of protein and ligands. The dodecahedron box was defined and filled with simple-point-charge water molecules by setting a minimal distance of 1.0 between the solute and the box (Berendsen & Marrink, 1993). Further the Na^+^ and Cl^−^ ions were added to neutralize the system. To relax the system the energy minimization was applied using steepest-descent minimization algorithm for all atoms so that the maximum force was no greater than 1,000 kJ^*x*^ mol^−1*x*^ nm^−1^ was. At the next stage the two-step equilibration was proceeded to equilibrate the ions and solvent around the protein. For the first equilibration the NVT (constant number of particles, volume, and temperature) ensemble within 100 ps (picosecond, or 10^−12^ of a second) was used to stabilize the temperature of the system at 300 K. Next the 100 ps NPT (constant number of particles, pressure, and temperature) equilibration used a coupling reference pressure of 1 bar to stabilize the system. Upon completion of the two equilibration phases, the system was well-equilibrated at the desired temperature and pressure. Finally, the 10 ns (nanosecond, or 10^−9^ of a second) MD simulation with a time step of 2 fs (femtosecond, or 10^−15^ of a second) had run. During the simulation, the leap-frog algorithm integrating Newton’s equations of motion was used for pressure control, long-range electrostatics were calculated by the particle-mesh Ewald method, all bond lengths were constrained by LINCS (Linear Constraint Solver) algorithm and the energy information and trajectory information were collected every 10 ps (Van Gunsteren & Berendsen, 1988; Essmann et al., 1995; Hess et al., 1997). As a postprocessing tool, Gromacs utilities were used for further analysis.

## Results

### Docking simulations

**Figure 2:**
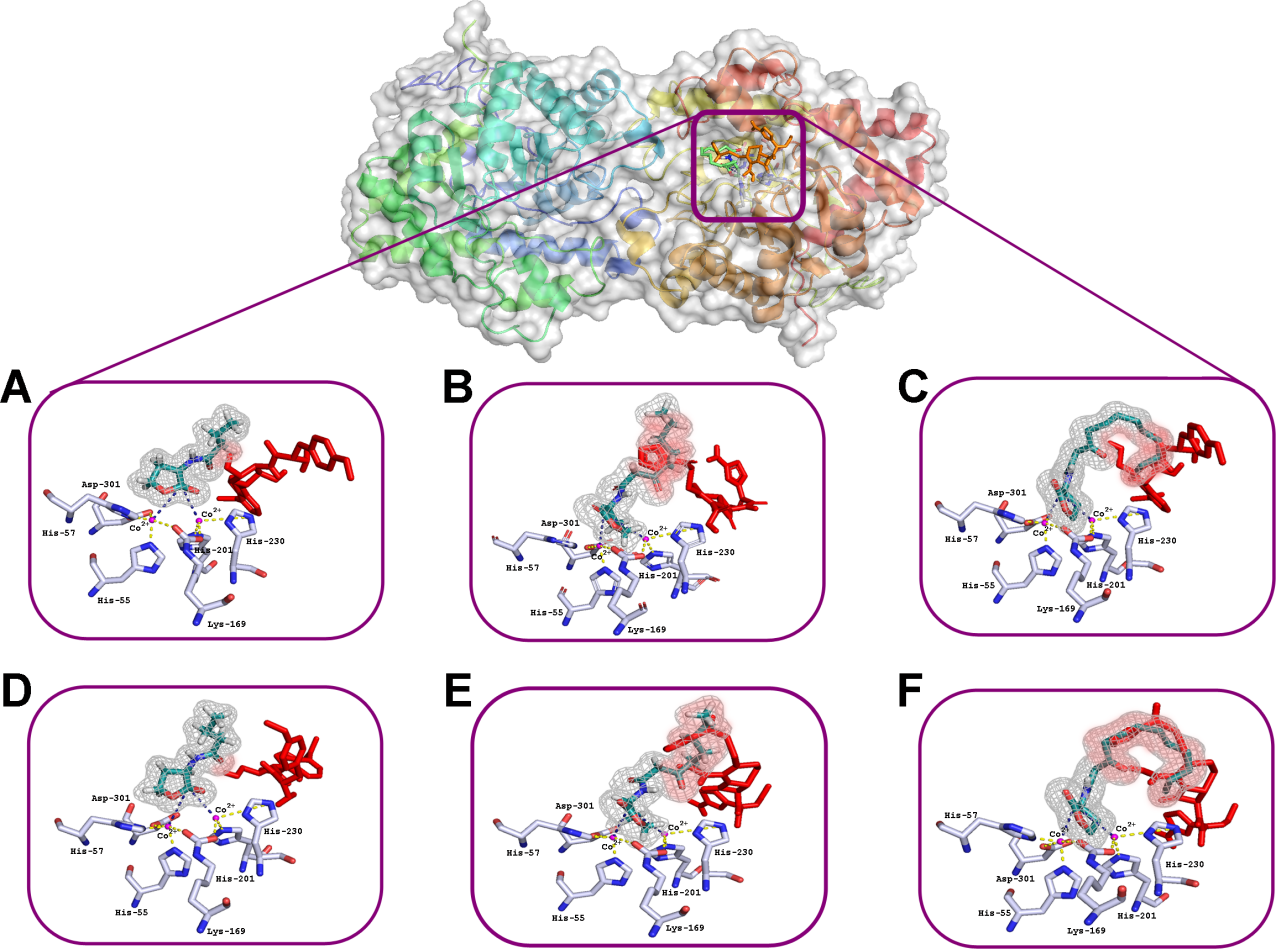
Results of computationally simulated interactions of AHLs with the active site of His_6_-OPH in the presence of puromycin (shown as red sticks). Interactions of C_4_- (A, D), C_8_- (B, E) and C_12_-containing AHLs (C, F) at pH 7.5 (A, B, C) and 10.5 (D, E, F). AHL molecules are shown as deep green sticks with grey mesh. His_6_-OPH active site residues and Co^2+^ ions are colored grey and violet, respectively. Interactions between lactone ring of AHLs and amino acid residues composing active site of His_6_-OPH are indicated by dashed lines. The intersection area of puromycin with AHLs is colored red.

**Figure 3:**
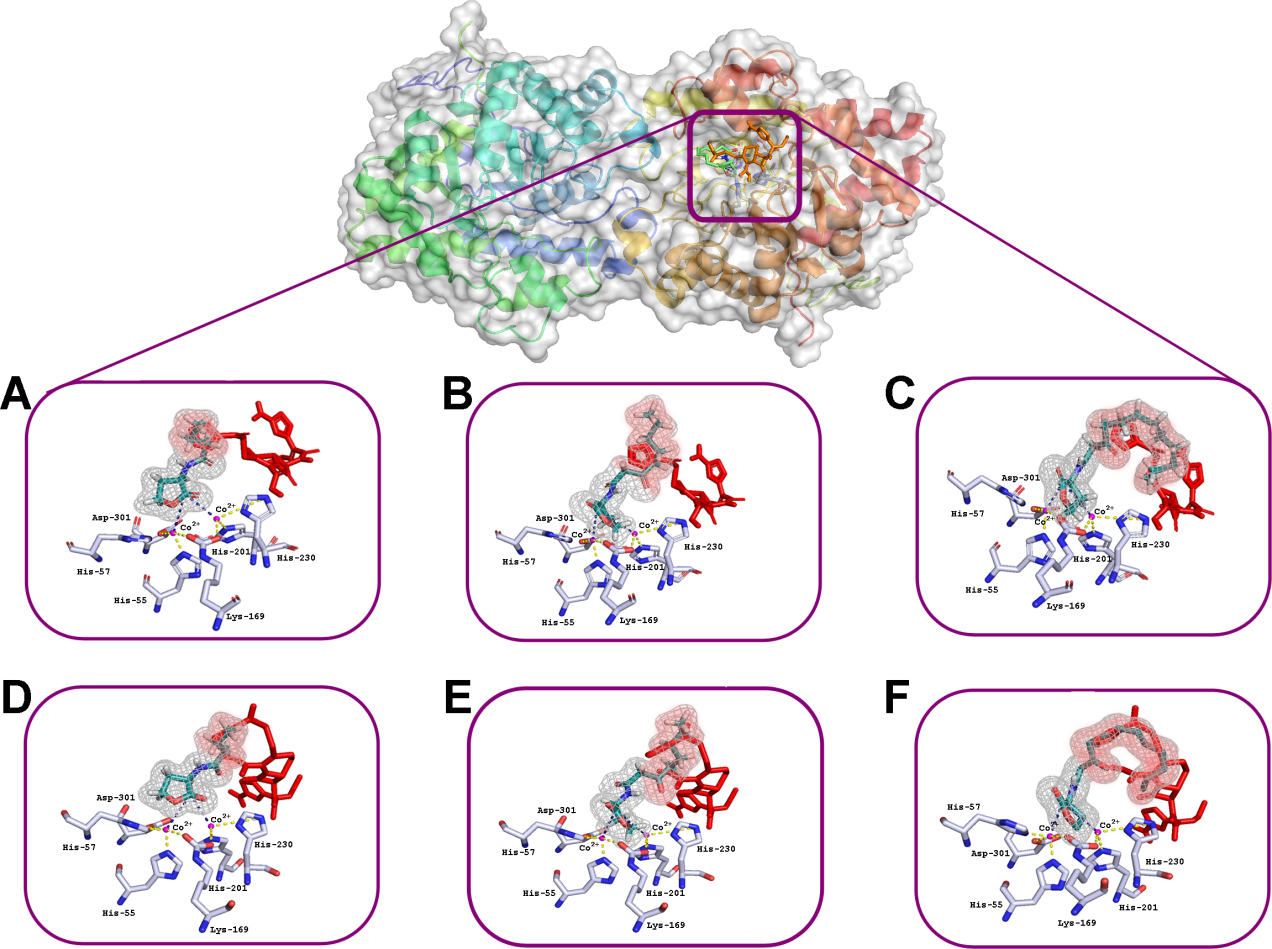
Results of computationally simulated interactions of AHLs with the active site of His_6_-OPH in the presence of ceftiofur (red stick). Interactions of C_4_–(A, D), C_8_-(B, E) and C_12_-containing AHLs (C, F) at pH 7.5 (A, B, C) and 10.5 (D, E, F). AHL molecules are shown as deep green sticks with grey mesh. His_6_-OPH active site residues and Co^2+^ ions are colored grey and violet, respectively. Interactions between lactone ring of AHLs and amino acid residues composing active site of His_6_-OPH are indicated by dashed lines. The intersection area of ceftiofur with AHLs is colored red.

The interactions of C4-HSL, C8-HSL and C12-HSL with the active sites of enzyme dimer molecules were simulated in the presence of puromycin and ceftiofur docked to the surface of His_6_-OPH at pH 7.5 (which corresponds to the physiological conditions of the antibiotics’ use) and 10.5 (which ensures the maximum catalytic activity of His_6_-OPH) (Votchitseva et al., 2006) (Figs. 2 and 3).

Small, individual differences were shown in the His_6_-OPH active sites when the relative position of the molecules of each of the antibiotics and the studied AHLs were taken in different combinations. The possibility of the intersection of AHLs with both antibiotic molecules was revealed when entering the enzyme’s active site. Moreover, the increase of AHLs acyl chain length was accompanied by an increase in the probability of its intersection with the antibiotics.

The binding energies of various ligands (AHLs and antibiotics) with the His_6_-OPH dimer surface were calculated (Table 1).

**Table 1:** Binding energy of ligands (antibiotics and AHLs) with His_6_-OPH at different pH. Scoring function named affinity (Trott & Olson, 2010) is an estimated minimum of potential energy during electrostatic and hydrophobic interactions, and hydrogen bonding between ligand (antibiotic or AHL) and receptor (enzyme).

Ligand	pH	Affinity, (kJ^*x*^ mol^−1^)				*p* value
		Mean	Median	**Range**	
Puromycin	7.5	−29.9	−30.1 ± 0.8	−29.3	−30.5	0.317
	10.5	−30.3	−30.1 ± 1.0	−29.3	−31.0	
Ceftiofur	7.5	−29.7	−29.3 ± 1.2	−29.0	−30.1	0.837
	10.5	−29.8	−29.5 ± 1.3	−29.0	−30.7	
C4-HSL	7.5	−21.8	−21.9 ± 1.8	−23.1	−20.7	0.170
	10.5	−22.5	−22.7 ± 0.8	−22.7	−22.0	
C8-HSL	7.5	−23.2	−23.2 ± 2.3	−23.9	−22.5	0.206
	10.5	−23.9	−23.4 ± 1.3	−24.5	−22.8	
C12-HSL	7.5	−23.8	−23.7 ± 1.2	−24.4	−23.2	0.360
	10.5	−23.5	−23.0 ± 1.1	−24.3	−22.7	

Statistically significant differences (according to one-way ANOVA, *p* = 0.003) were observed between the values obtained for C4-HSL and C12-HSL, whereas there were no statistically significant differences between the values of the other groups (*p* ≥ 0.100). It has also been shown that the binding energies of AHLs with His_6_-OPH were slightly higher in comparison with the binding energies of antibiotics with the enzyme. Therefore, the binding of antibiotics to the enzyme’s active site seems to be an energetically more beneficial process, which means that when competing with AHLs, they must displace the latter from the His_6_-OPH dimer active sites.

### Binding properties

**Figure 4:**
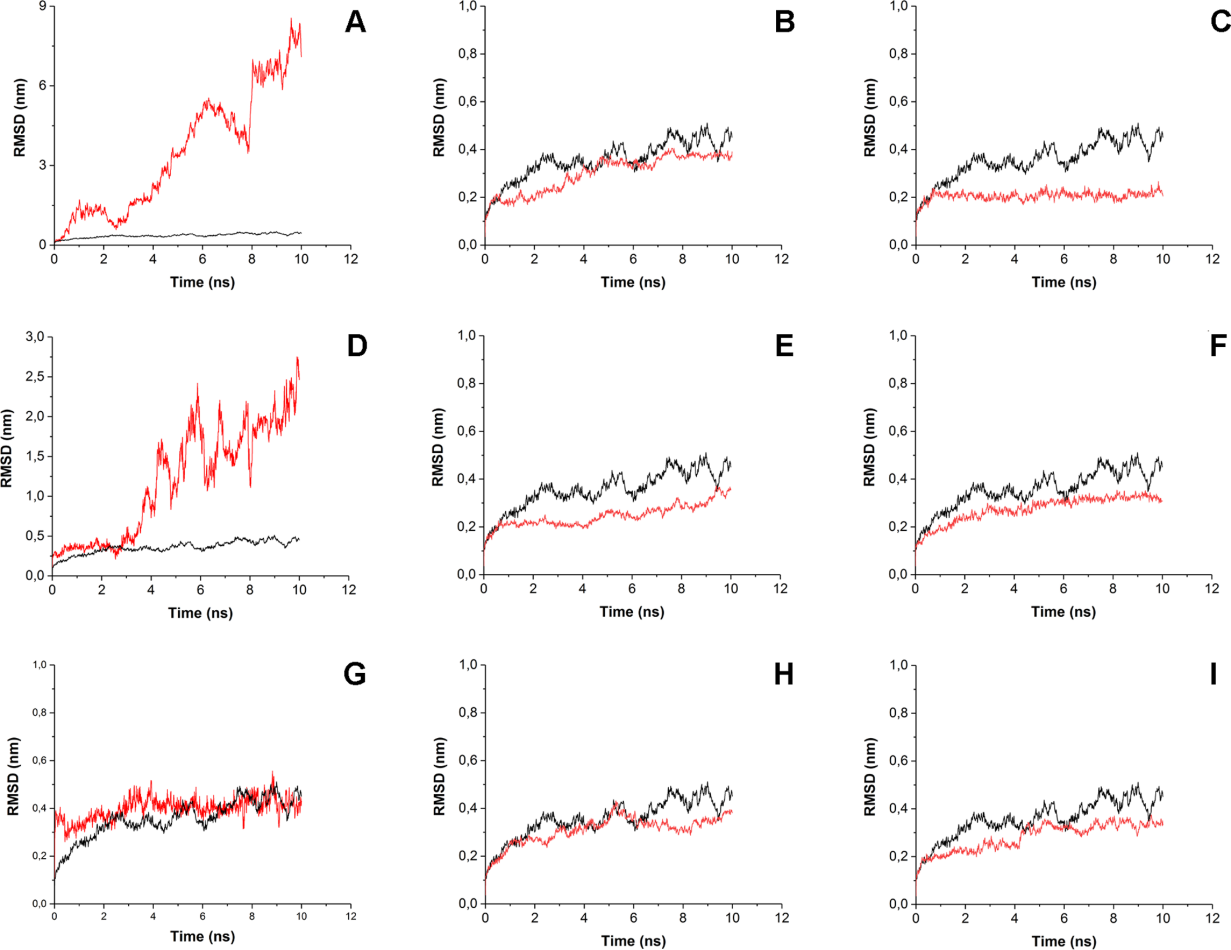
Root-mean-square deviation (RMSD) of enzyme-substrate complexes. Root-mean-square deviation (RMSD) of enzyme-substrate complexes of His_6_-OPH with C4-, C8- and C12-HSLs in the absence (A, D, G) and presence of puromycin (B, E, H) and ceftiofur (C, F, I) (red). Complex of His_6_-OPH with paraoxon was used as a reference (black).

**Figure 5:**
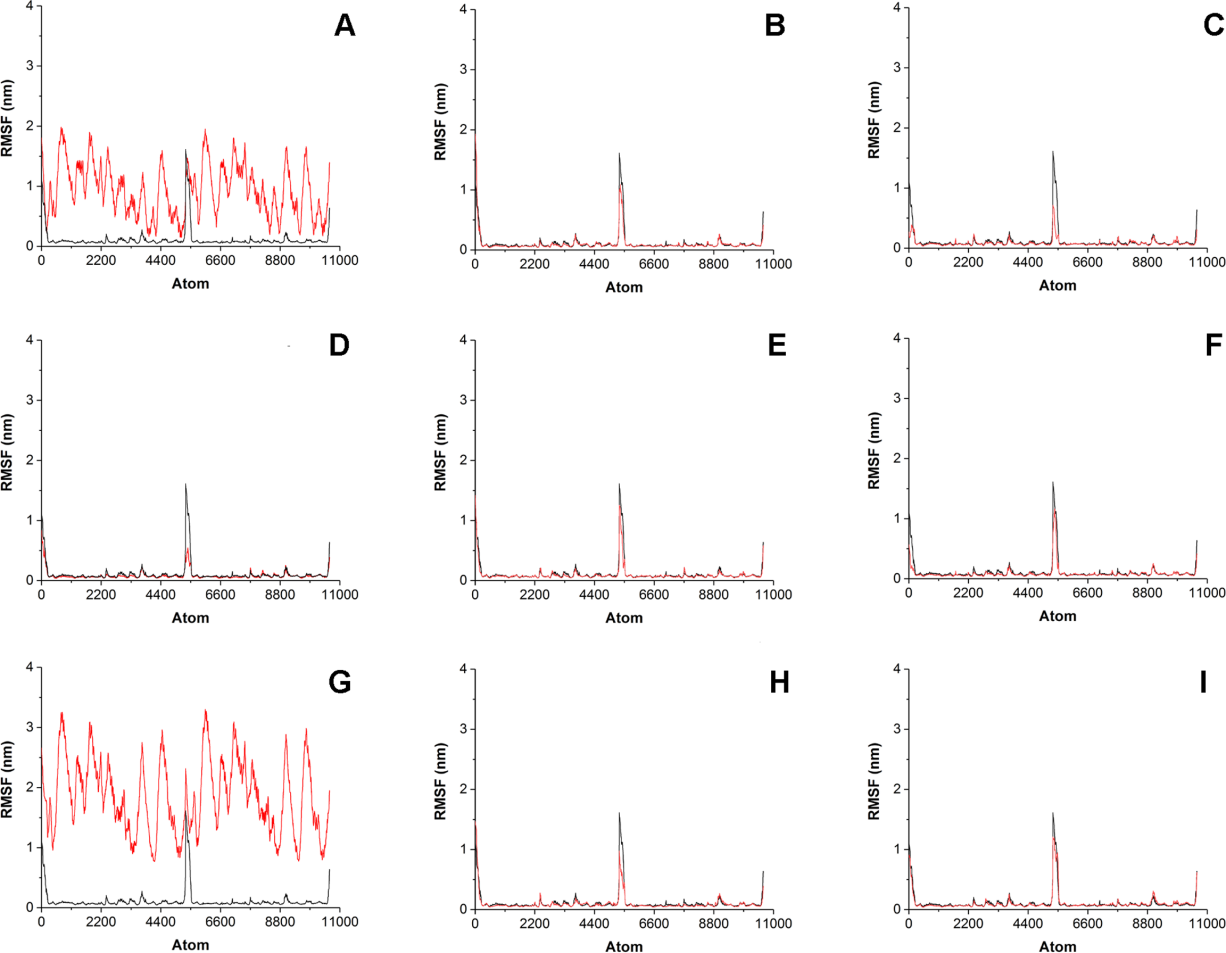
Root-mean-square fluctation (RMSF) of enzyme-substrate complexes. Root-mean-square fluctation (RMSF) of enzyme-substrate complexes of His_6_-OPH with C4-, C8- and C12-HSLs in the absence (A, D, G) and presence of puromycin (B, E, H) and ceftiofur (C, F, I) (colored red). Complex of His_6_-OPH with paraoxon was used as a reference (colored black).

To estimate how does the antibiotics binding to the surface of His_6_-OPH affect the behavior of enzyme-substrate interactions, the root-mean-square-deviation (RMSD) and the root-mean-square-fluctation (RMSF) analysis were performed (Figs. 4 and 5). Same parameters established for His_6_-OPH in enzyme-substrate complex with paraoxon being pesticide and the regular substrate of the enzyme (Votchitseva et al., 2006; Lyagin & Efremenko, 2019) were used as a reference in this analysis.

It is known that the lower the RMSD value, the better ligands position is fixed in the protein-ligand complex. According to Fig. 4 the steadiness of substrate position in the complex of His_6_-OPH with AHL increases from C4-HSL to C12-HSL. In all cases, the combination of His_6_-OPH with antibiotic led to an increase in the steadiness of AHLs position in enzyme-substrate complexes.

Significant fluctuations were observed for AHL atoms in enzyme-substrate complexes in the absence of antibiotics, except C8-HSL (Fig. 5). Evidently, combination of antibiotics with the His_6_-OPH provides the fixation of the position of substrate molecule in the His_6_-OPH-AHL complexes.

To quantify the strength of the interaction between AHLs and His_6_-OPH in the absence and presence of antibiotics the interaction energies between enzyme and AHLs were calculated (Table 2).

**Table 2:** Effect of His_6_-OPH combination with antibiotics on the interaction energy of AHLs as substrates with the enzyme.

Enzyme or combinations	Interaction energy, kJ^*x*^ mol^−1^		
	S_1_^*^	S_2_	S_3_
His_6_-OPH	−25.05 ± 7.38	−57.51 ± 12.08	−153.62 ± 8.60
His_6_-OPH/A_1_^**^	−72.27 ± 11.97	−227.60 ± 15.01	−138.44 ± 7.58
His_6_-OPH/A_2_	−86.60 ± 3.06	−109.52 ± 12.18	−129.67 ± 6.52

**Notes.**

* S_1_, S_2_, S_3_ are C4-, C8- and C12-HSLs, respectively.

** A_1_, A_2_ are puromycin and ceftiofur, respectively.

As a result, the highest interaction strength was observed for His_6_-OPH-C8-HSL complex in the presence of pyromycin. The interaction strength of C12-HSL with all three tested enzymatic samples were high enough, whereas C4-HSL showed relatively low interaction strength.

To understand whether reaction of AHLs as substrates with His_6_-OPH in the presence of antibiotics affects the interactions between enzyme and antibiotics, the interaction energies of antibiotics with His_6_-OPH were calculated for His_6_-OPH/antibiotic-AHL systems (Table 3).

**Table 3:** Effect of enzyme-substrate binding on the interactions of His_6_-OPH with antibiotics.

Substrate	Interaction energy, kJ^*x*^ mol^−1^	
	His_6_-OPH/A_1_^**^	His_6_-OPH/A_2_
w/o substrate	−76.22 ± 5.34	−81.32 ± 7.44
S_1_^*^	−94.54 ± 5.52	−130.52 ± 28.32
S_2_	−11.73 ± 6.86	−139.09 ± 11.08
S_3_	−55.70 ± 6.38	−61.79 ± 11.83

**Notes.**

* S_1_, S_2_, S_3_ are C4-, C8- and C12-HSLs, respectively.

** A_1_, A_2_ are puromycin and ceftiofur, respectively.

The results presented in the Table 3 showed that ceftiofur binds with His_6_-OPH stronger than puromycin in all cases. Formation of the His_6_-OPH-C4-HSL complex in the presence of antibiotics led to increased interaction strength between enzyme and antibiotic, while complex with C12-HSL resulted in its decrease. In case of enzyme reaction with C8-HSL the interaction strength inside the combination His_6_-OPH/ceftofur was increased and significantly decreased in His_6_-OPH/puromycin.

## Discussion

It is known that the closer and more accessible the lactone ring of the hydrolyzable AHLs is oriented to the active site and to the Co^2+^ ions that participate in the catalytic act of the enzyme, the higher the probability of the occurrence of effective enzymatic catalysis. In addition, the acyl chain of AHLs should be localized outside of the active site.

The docking of AHLs to the His_6_-OPH dimer surface in the presence of some antibiotic (Figs. 2 and 3) showed that the latter can directly influence the orientation of the AHLs in the enzymatic active site and that the longer the acyl chain of AHLs, the greater the observed effect. When the molecules of both ligands are adjacent to the enzymatic active site, the antibiotics competes with the AHLs for localization in the same place, thereby affecting the efficiency of AHLs hydrolysis.

RMSD and RMSF were used to evaluate the interactions of the His_6_-OPH with substrates (various AHLs) in the presence and absence of different antibiotics. The results of RMSD and RMSF analysis showed that the combination of His_6_-OPH with both puromycin and ceftiofur leads to a significant fixation of the AHLs position in coordination with the active center of His_6_-OPH in enzyme-substrate complexes. Moreover, the longer the acyl chain of AHLs, the more stable it appears (Fig. 4).

It is known that His_6_-OPH exhibits the highest catalytic activity against paraoxon among the other organophosphorus substrates for the action of this enzyme (Lyagin & Efremenko, 2019). Based on this, the enzyme-substrate complex of His_6_-OPH with paraoxon was used as a “reference standard” when comparing the results of RMSD and RMSF analysis obtained for His_6_-OPH-AHL complexes. It has been established, that the combination of His_6_-OPH with antibiotics leads to the same efficient hydrolysis of AHLs as paraoxon.

To assess how the presence of puromycin or ceftiofur affects the behavior of the enzyme-substrate interactions, the values of interaction energy between AHLs and His_6_-OPH in the absence and presence of antibiotics were calculated. As a result, it was revealed that from a thermodynamic point of view, the longer the acyl chain of AHLs, the more favorable and, as a result, the more effective is a formation of the enzyme-substrate complex. The use of His_6_-OPH/antibiotic combinations has proven thermodynamically to be more efficient for C4-HSL and C8-HSL hydrolysis as compared to C12-HSL, for which the most effective interaction was observed with native His_6_-OPH. By that, the results of MD simulations confirmed the assumptions made on the basis of molecular docking: the combination of His_6_-OPH with antibiotics leads to a direct influence on the enzyme-substrate binding and, therefore, on the efficiency of the enzymatic reaction. Moreover, the longer the acyl chain of AHLs, the pronounced is the affect of the antibiotic on the catalytic reaction, and the less effective is the interaction between the enzyme and the AHLs.

## Conclusions

The results obtained in this study indicate that puromycin and ceftiofur, being combined with His_6_-OPH, increase the steadiness of AHLs position in the enzyme-substrate complexes, but at the same time tested antibiotics reduce the effectiveness of enzymatic hydrolysis of long-chain AHLs as compared to the native enzyme. Therefore, this should be taken into account when creating a possible therapeutic composition based on combining antibiotics with His_6_-OPH.

Based on the data obtained, it can be concluded that the computational methods such as molecular docking and molecular dynamic simulations can be further utilized for effective selection of possible partners among differently used antibiotics for enzymes with lactonase activity, like His_6_-OPH, in order to create maximally efficient preparations with improved action against antibiotic-resistant bacterial cells.

##  Supplemental Information

10.7717/peerj.7684/supp-1 Figure S1 Structures of the His_6_-tag predicted using I-TASSER server Designation: green coils –models of the tag alone; yellow, red and blue coils –models of the tag within His_6_-OPH. The most similar structures are emphasized with blue (model #1) and red (model #3) color and are covered with molecular surface.

10.7717/peerj.7684/supp-2 Figure S2 Supposed localization of the domains for binding puromycin at pH 7.5 (A) and 10.5 (B) The two subunits of the His_6_-OPH homodimer are colored differently (grey and dark grey). Atoms on the surface of His_6_-OPH located within 4 Å of any atom of puromycin, as well as the corresponding molecular surface, are colored yellow (A) or red (B). Entrances to the active sites of the His_6_-OPH dimer are highlighted with red (A) or yellow (B) boxes. Purple boxes show a His_6_-OPH dimer molecule when it is viewed from below (II) and above (III) (relative to the side where the active sites are located).

10.7717/peerj.7684/supp-3 Figure S3 Supposed localization of the domains for binding ceftiofur at pH 7.5 (A) and 10.5 (B) The two subunits of the His_6_-OPH homodimer are colored differently (grey and dark grey). Atoms on the surface of His_6_-OPH located within 4 Å of any atom of ceftiofur, as well as the corresponding molecular surface, are colored yellow (A) or red (B). Entrances to the active sites of the His_6_-OPH dimer are highlighted with red (A) or yellow (B) boxes. Purple boxes show a His_6_-OPH dimer molecule when it is viewed from below (II) and above (III) (relative to the side where the active sites are located).

10.7717/peerj.7684/supp-4 File S1 Protein Data Bank (PDB) files for docked ligands XYZ coordinates of antibiotics (puromycin and ceftiofur) and AHLs (C4-, C8- and C12-HSL) docked to the surface of His_6_-OPH.

## Additional Information and Declarations

### Data Availability

The following information was supplied regarding data availability: The raw data are provided in the Supplemental Files.
