# Supplementary figures and images for: Simultaneous molecular docking of different ligands to His6-tagged organophosphorus hydrolase as an effective tool for assessing their effect on the enzyme
Source: PeerJ. 2019 Sep 12;7:e7684. doi: 10.7717/peerj.7684 (PMC6745196; doi:10.7717/peerj.7684)

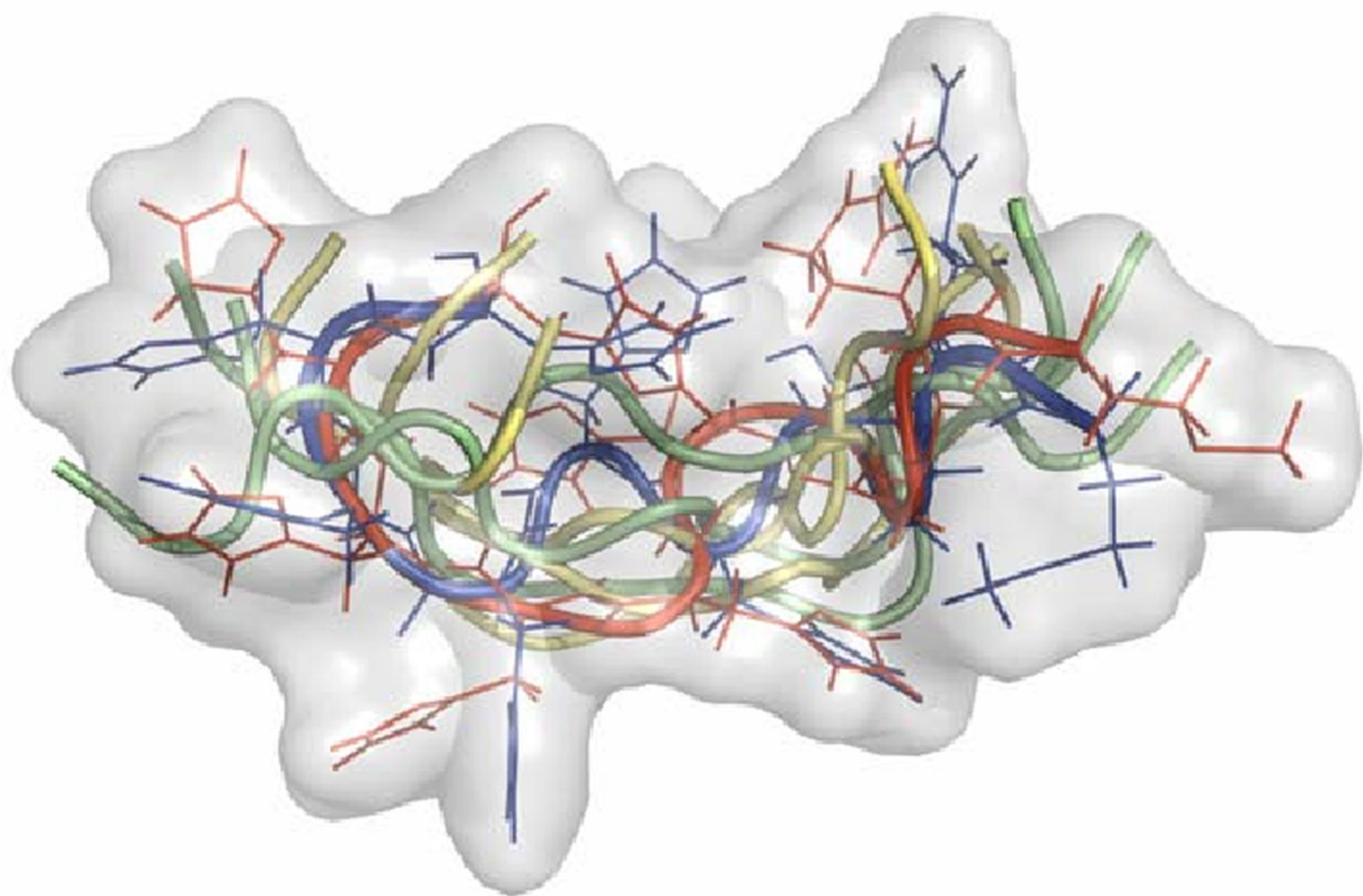

Supplement: Figure S1 — Designation: green coils –models of the tag alone; yellow, red and blue coils –models of the tag within His6-OPH. The most similar structures are emphasized with blue (model #1) and red (model #3) color and are covered with molecular surface. [file peerj-07-7684-s001.pdf]

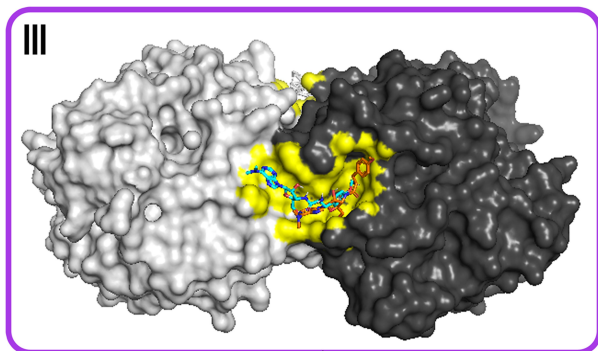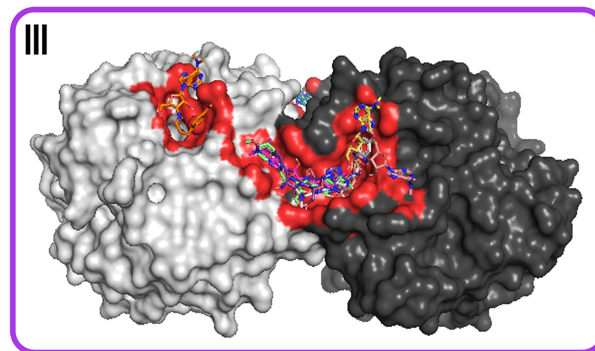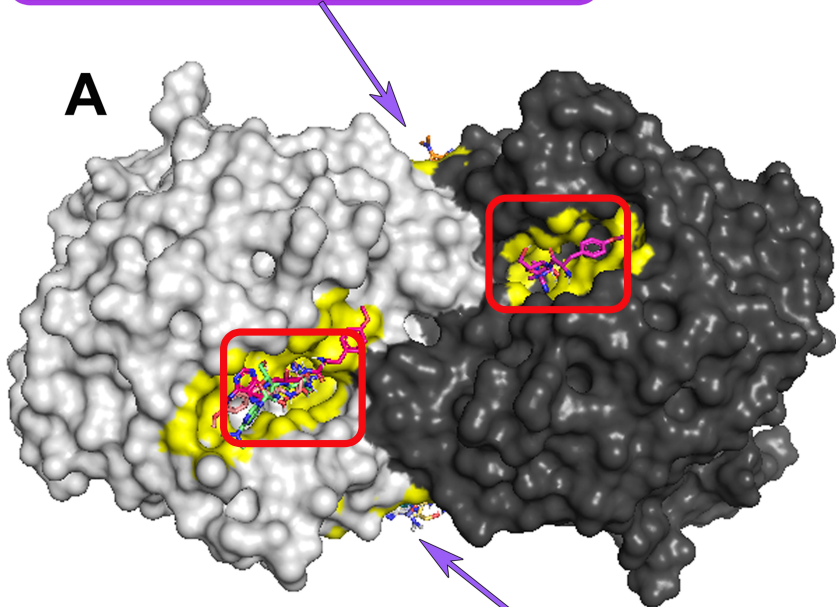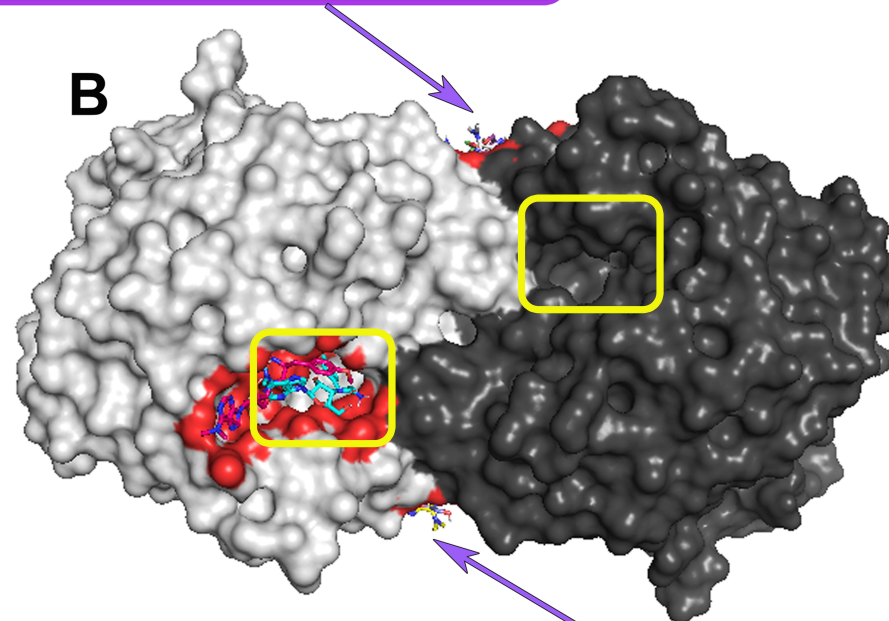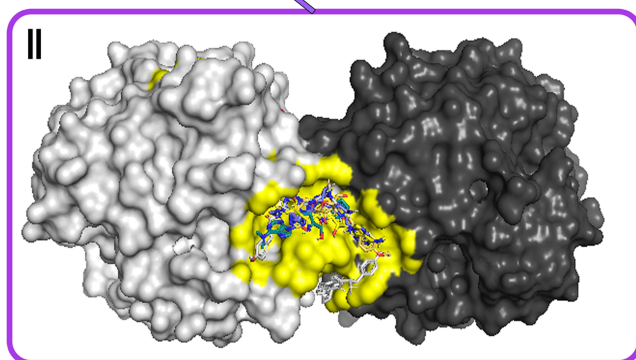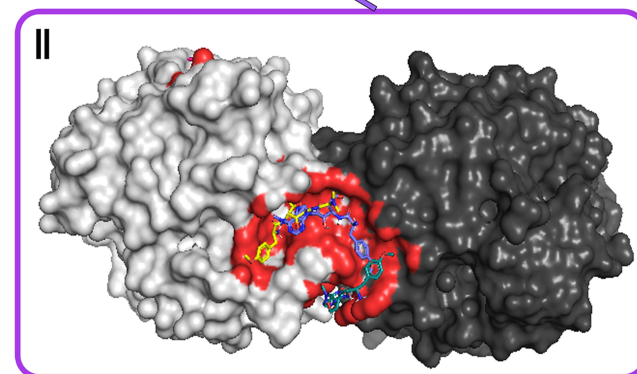

Supplement: Figure S2 — The two subunits of the His6-OPH homodimer are colored differently (grey and dark grey). Atoms on the surface of His6-OPH located within 4 Å of any atom of puromycin, as well as the corresponding molecular surface, are colored yellow (A) or red (B). Entrances to the active sites of the His6-OPH dimer are highlighted with red (A) or yellow (B) boxes. Purple boxes show a His6-OPH dimer molecule when it is viewed from below (II) and above (III) (relative to the side where the active sites are located). [file peerj-07-7684-s002.pdf]

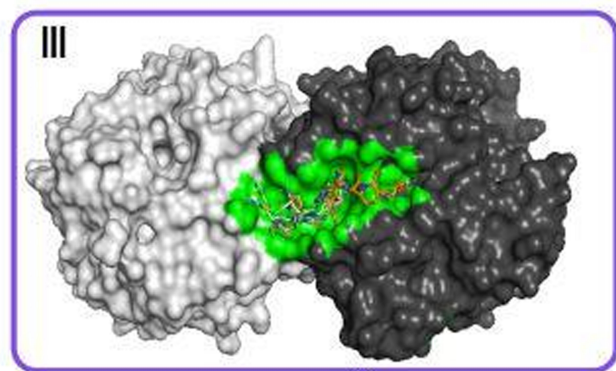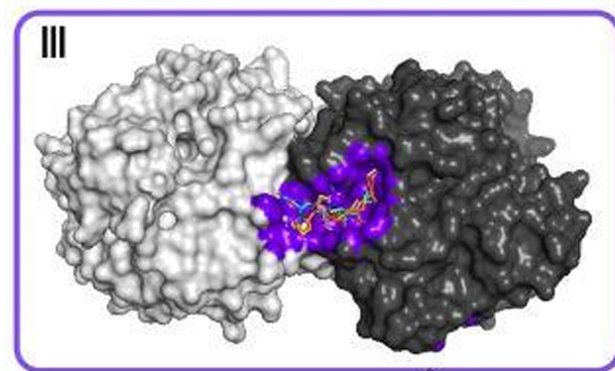

A

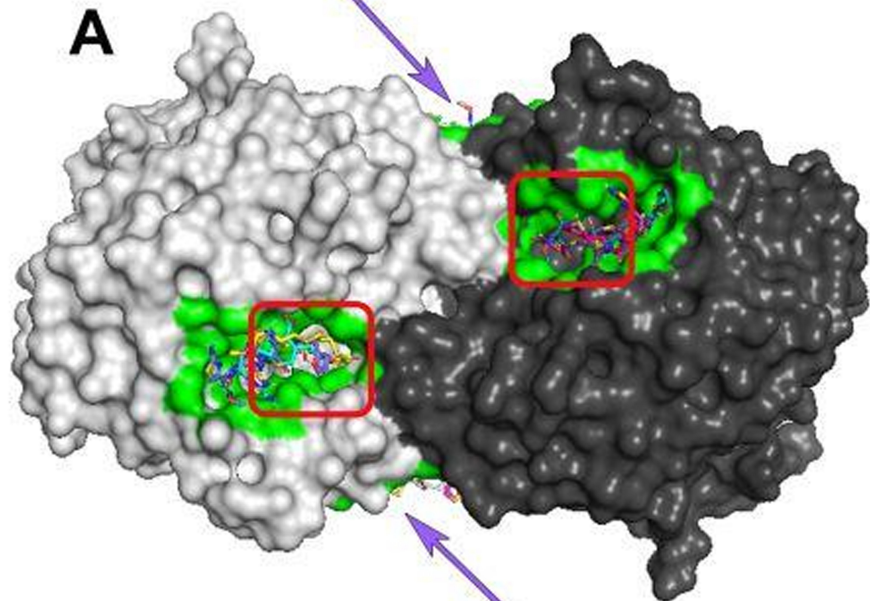

Б

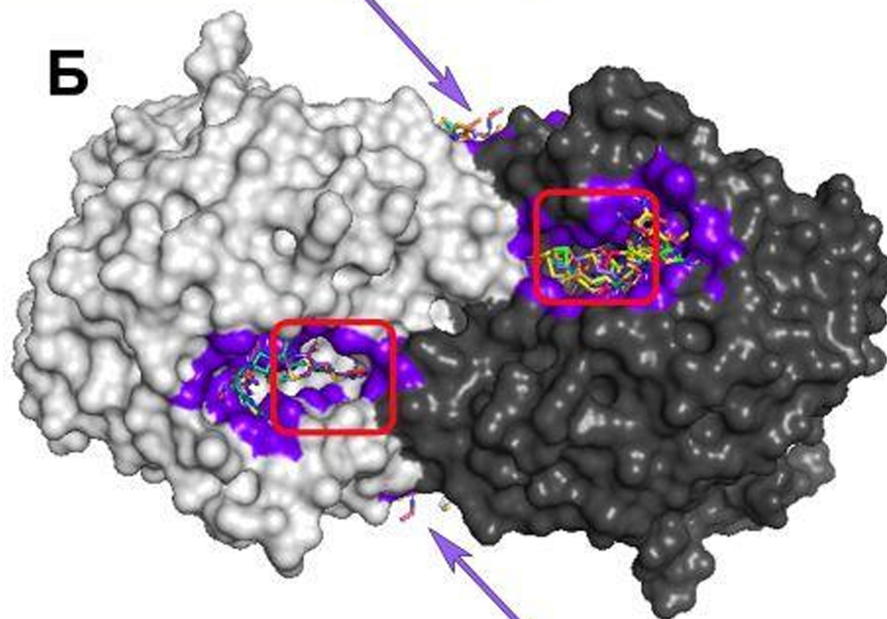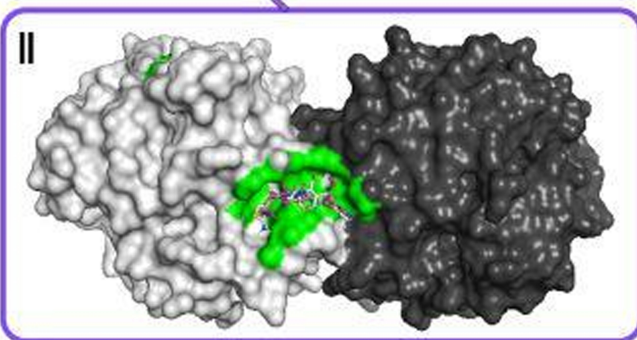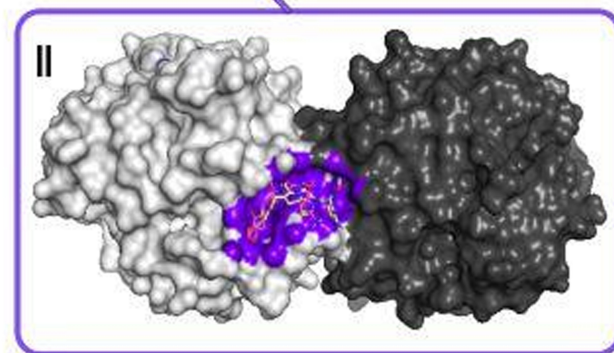

Supplement: Figure S3 — The two subunits of the His6-OPH homodimer are colored differently (grey and dark grey). Atoms on the surface of His6-OPH located within 4 Å of any atom of ceftiofur, as well as the corresponding molecular surface, are colored yellow (A) or red (B). Entrances to the active sites of the His6-OPH dimer are highlighted with red (A) or yellow (B) boxes. Purple boxes show a His6-OPH dimer molecule when it is viewed from below (II) and above (III) (relative to the side where the active sites are located). [file peerj-07-7684-s003.pdf]
